# Cosmetic Surgery: Regulatory Challenges in a Global Beauty Market

**DOI:** 10.1007/s10728-017-0339-5

**Published:** 2017-02-28

**Authors:** Danielle Griffiths, Alex Mullock

**Affiliations:** 1 School of Law, University of Sussex, Sussex, UK; 2 School of Law, University of Manchester, Manchester, UK

**Keywords:** Cosmetic surgery, Regulation, Criminal law

## Abstract

The market for cosmetic surgery tourism is growing with an increase in people travelling abroad for cosmetic surgery. While the reasons for seeking cosmetic surgery abroad may vary the most common reason is financial, but does cheaper surgery abroad carry greater risks? We explore the risks of poorly regulated cosmetic surgery to society generally before discussing how harm might be magnified in the context of cosmetic tourism, where the demand for cheaper surgery drives the market and makes surgery accessible for increasing numbers of people. This contributes to the normalisation of surgical enhancement, creating unhealthy cultural pressure to undergo invasive and risky procedures in the name of beauty. In addressing the harms of poorly regulated surgery, a number of organisations purport to provide a register of safe and ethical plastic surgeons, yet this arguably achieves little and in the absence of improved regulation the risks are likely to grow as the global market expands to meet demand. While the evidence suggests that global regulation is needed, the paper concludes that since a global regulatory response is unlikely, more robust domestic regulation may be the best approach. While domestic regulation may increase the drive towards foreign providers it may also have a symbolic effect which will reduce this drive by making people more aware of the dangers of surgery, both to society and individual physical wellbeing.

## Introduction

Travelling abroad for medical reasons is not a new phenomenon. Victorian travellers who travelled to ‘take the waters’ or to breathe fresh sea or mountain air for health purposes helped to lay the foundations of modern tourism [25]. More recently, however, a new type of medical tourist has emerged. Cosmetic surgery tourism is a fast growing market and in a global society the reasons for seeking cosmetic surgery abroad may vary significantly. Elite consumers may seek the services of the very best cosmetic surgeons in the world, while those seeking more extreme or even risky procedures may simply be unable to get what they want in the UK. The most common reason for travelling abroad for cosmetic surgery rather than remaining in one’s home country to access domestic services, however, is financial. Cosmetic surgery can be considerably less expensive outside the UK, with a recent report suggesting that popular procedures such as ‘nose jobs’ (rhinoplasty) and breast augmentation may be approximately £2000 cheaper in the Czech Republic and Poland than in the UK.^1^ Consequently, the increasing appetite for affordable cosmetic surgery has led to a growth in such surgical holidays. And although the services that this new breed of traveller is seeking are indeed medical—hopefully involving qualified surgeons working within private hospitals—the consequences of travelling abroad for cosmetic surgery, within a highly commercial and poorly regulated industry, may be far from medically beneficial.

In this paper we explore some of the concerns over the rapidly expanding global market in cosmetic surgery before considering the challenges of attempting to regulate the cosmetic surgery market. Data available on cosmetic surgery is patchy; there are small-scale sociological studies, surveys conducted by certain interested parties including professional organisations of plastic surgeons and medical defence organisations who represent surgeons in legal proceedings, and there is some data kept by the NHS. Such lack of evidence means that we do not have truly accurate data on the incidence of procedures in the UK and elsewhere or the resulting harm. Yet there is a lot we can glean from the data that does exist, although in using the data in this paper we do not claim our conclusions are representative.

We begin by briefly reviewing the evidence and recent developments pertaining to cosmetic surgery in the UK, considering what is driving the increase in such surgery and what cultural and physical harms are resulting, as well as how the law might respond. We then situate the domestic evidence within the global phenomenon of cosmetic surgery. We assess the evidence and apparent risks of cosmetic surgery tourism and the implications for the UK and the NHS in particular. We ask whether such foreign surgery should necessarily, and always, be regarded as more dangerous or whether the domestic market is raising concerns in order to protect itself. Either way we argue that the global trends are harmful but while a domestic response is crucial, its power to reduce harm is restricted by the drive for cheaper surgery in certain foreign countries.

In the context of global dominant beauty norms and the cultural and individual physical harms that they bring, we conclude by asking what the role of the law should be in this context. Is a global regulatory response possible? If the potential for global regulation is extremely limited we should consider how else to address the harms at stake and begin by addressing the inadequacy of domestic regulation. In response to the concern that tighter domestic regulation will encourage cosmetic tourism we agree with McHale that ‘it is too easy to assume that globalisation means that resistance in the form of regulation is futile’ [21]. Consequently we argue that a domestic regulatory response that seeks to make cosmetic surgery provision subject to increased governance is necessary to combat the dual harms (cultural and physical) we identify. Firstly, tighter regulation would make commercial provision safer for the consumer. Secondly, it would send a clear message about the potential dangers of cosmetic surgery in order to influence societal perceptions and resist the increasing normalisation of surgery as beauty treatment.

## The Growth and Normalisation of Cosmetic Surgery Within the UK: Difference and Sameness

Plastic surgery is surgery undertaken for the purposes of altering the appearance of a patient. There are two subfields within the broader medical practice of plastic surgery. Reconstructive surgery is defined as work that seeks to ‘repair, catastrophic, congenital, or cancer-damage deformities and is seen as restorative of a somehow damaged appearance, whereas cosmetic surgery is defined as ‘entirely elective’ work that is seen as purely as enhancement of appearance [3]. And while all surgery carries risks, the risks of cosmetics surgery should be more carefully weighed because they cannot be justified on health grounds but rather, the serious risks are undertaken for purely aesthetic reasons. This aesthetic rather than therapeutic purpose, as we have argued elsewhere [12], changes the nature of the risk/benefit analysis and, compared to medically necessary surgery, the risk/benefit analysis of cosmetic surgery should necessitate a more precautionary approach.

Over the past few decades, societal attitudes to cosmetic surgery have evolved quite dramatically. Undergoing surgery as a beauty enhancing treatment has become a lifestyle choice for increasing numbers of people, with a significant increase in people electing to undergo such procedures. According to the British Association of Aesthetic Plastic Surgeons (BAAPS), 50,122 cosmetic procedures were performed in 2013, a rise of 17% from 2012 [5]. The cosmetic surgery industry was worth £750 m in the UK in 2005, £2.3bn in 2010, and is forecast to reach £3.6bn by 2015 [10]. As Sir Bruce Keogh’s review of the industry recently reported, rising demand for cosmetic enhancement has been driven by a number of socio-economic and technological factors, leading to the normalisation of serious and potentially harmful cosmetic interventions [17]. Keogh’s report and other evidence shows that while surgery was once undertaken discreetly, now many more people will admit to it and even celebrate it. The media, social media, celebrity endorsement and advertising have been central to this normalisation [21].

Looking at gender issues within this phenomenon, we can see that while men are opting for surgery in greater numbers than ever before, it is still women who predominantly undergo such treatments [17]. The early feminist position was that women who have cosmetic surgery are victims of a patriarchal culture and beauty industry that pressurises them into making themselves more sexually desirable to men. Within this initial feminist response, women undergoing surgery are viewed as passive victims of a patriarchal system who are capitulating to their own sexual objectification. Subsequently this view was challenged by other feminists who emphasised questions of choice, autonomy and self-determination. Most famous in this respect is Kathy Davis’s work, which portrays women as active agents, carefully negotiating and controlling their surgeries rather than being mere puppets of patriarchy. However, this account has been critiqued for overemphasising women’s agency. Bordo, for example, states that Davis presents the self as an ‘authentic and personal reference point untouched by external values and demands or relations with others [4]. The third position that has evolved, and which accords more with our position, regards the motivation for cosmetic surgery as neither fully internal nor external but rather an intersubjective and embodied process that takes place in a consumerist environment [22].

Placing aesthetic non therapeutic surgery within an intersubjective and consumerist context shifts the focus away from the disembodied individual cosmetic surgery patient that has dominated much previous research (which portrays her as either the victim of patriarchy or free autonomous agent), and allows us to take into account agency and choice within constrained cultural context. Bodies are active and reflexive but also heavily influenced by other bodies and gendered and racialised norms [26]. In this position, cosmetic surgery is approached as ‘a purchase, characterised by the rhetorics of fashion, consumption and self-presentation rather than medical or psychological necessity’ [22]. Here then for us ‘consumption of cosmetic surgery is a strategic act on the part of individuals who are rational and intelligent but who reside in a structural context where class, gender and race determine action’ [22]. Latham’s work has also been informative here [19]. Following her review of the feminist literature Latham advocated for a ‘third way’, which promotes a relational approach to autonomy and which seeks to address the conflicting feminist concerns through more precautionary yet pragmatic regulation.

Also significant is the fact that when we talk about consumers of surgery (both nationally and globally), we are talking about aged, classed and raced women. While surgery has become normalised, and global norms can be discerned, women are differently placed in relation to this normalisation in the UK and globally according to the cultural and social environment in which they reside. For example, in relation to social class, as Jacqueline Sanchez Taylor has recently noted ‘we are not all the same kind of makeover citizens, nor do we all experience the same pressure to conform to the same patriarchal ideals of feminine beauty.’ Some women, middle class academics for example, can (usually) do their gender without undergoing invasive procedures, they will not be dishonoured by their lack of attention to the kind of beauty and fashion regimes which are important to some other women and, in fact the reverse is often true, within an environment where low key performances of gender are typically more highly valued and respected [24].

In an ESRC study ‘Sun, Sea, Sand and Silicone’ women from different countries who were undergoing foreign cosmetic surgery were tracked [14]. Cultural differences in procedures the women wanted undertaken and why they were undertaking them were stark. For example, 44% of the British sample and 66% of the Australian sample were travelling abroad for breast augmentations. This procedure was absent for the sample of Chinese women who were travelling to South Korea for surgery, for whom eyelid, jawbone and nose jobs made up 88% of the total. The UK women were defined as mostly working class women, while the Chinese patients were mostly middle class women who sought better quality surgery than that which is available in China.

Sanchez Taylor has observed that:

[W]e may all live as engendered beings in a patriarchal society(ies), and may all metaphorically be cosmetic surgery recipients, we also have different class, age, sexual and racialized identities (as well as being differentiated along the lines of able-bodiness/disability) and so stand in different relations to outward displays of gender and are positioned differently in relation to contemporary discourses and practices of cosmetic surgery [24].

Recognising such difference is thus essential when we look at cosmetic surgery because not all women stand in the same relationship to beauty norms and the pressures to have surgery. While certain global beauty norms may be evident, such as the desire to look young, there are still key differences. Despite such differences, however, it is true to say that generally women share many of the pressures and for women who do turn to cosmetic surgery, similar risks may be apparent. Indeed, when we consider the potential risks and real harm that such surgery involves, both physically and arguably also culturally, we can see that many of the concerns are universal.

## Harm and Risk in UK Cosmetic Surgery

There are firstly the risks of what Jean McHale has described as ‘normalising perfection’ as well as pathologising imperfection [21]. Cosmetic surgery reinforces and heightens concern with body image and culturally prescribed standards of beauty, contributing to a youth culture that distains aging and the elderly and upholds culturally specific standards of beauty. It also promotes inequality between those who have the resources to purchase an enhanced appearance and those who don’t. As McHale has asked, will the ‘cosmetically unenhanced become an effectively unemployable underclass?’ [21]. While women are differently placed in relation to *having* to conform to standards of beauty and surgical enhancement, none are untouched by being placed in relation to these standards and even those women who do not feel beholden to explicit gender performances (or even surgery for economic or cultural capital) may be discredited in wider society for not doing so.

The physical risks of cosmetic surgery have most starkly and recently been illuminated by the PIP (Poly Implant Prothese) breast implant scandal, which resulted in global outrage after the French implant company, PIP, were found to have used industrial grade silicon in their product.^2^ More generally, data on harm in cosmetic procedures is scarce but some information is available from medical negligence claims. According to a major analysis of cosmetic surgery done by the Medical Defence Union (MDU), growing numbers of patients are suing cosmetic surgeons over mistakes during operations designed to improve their appearance.^3^ Data from this analysis shows that negligence claims concerning breast surgery, facelifts, eyelid operations, nose reductions, and weight-loss procedures account for 80% of claims stemming from cosmetic surgery and damages of more than £500,000 were paid out over a five year period. The MDU state that cosmetic surgery negligence claims are successful in 45% of cases, compared with 30% of medical negligence claims in general. This success rate would suggest that when harm occurs from cosmetic surgery, the presence of negligent behaviour is more often clearer and easier to prove than when harm occurs from medically necessary surgery. The reasons for this are unknown, however, we suggest that this might be at least partly because the surgery is not carried out for therapeutic reasons and so the resulting harm, to a victim who was (presumably) previously in good health, is an obvious sign that mistakes have been made. Additionally, the lack of regulation has meant that within the private market for cosmetic surgery, too often poorly qualified surgeons are undertaking procedures for which there has been an inadequate consent process coupled with inadequate consideration of the patient’s health and well being, which further heightens the usual risks associated with invasive surgery [17]. For this reason, as we have argued elsewhere [12], the unquestioning assumption that non-therapeutic cosmetic surgery is justified under the medical exception to the (English) criminal law as ‘proper medical treatment’, has led to a complacent approach to regulation that requires urgent attention.

In response to the Keogh Review, there have been some developments but not enough to fully protect the cosmetic surgery consumer. Consequently, the president of BAAPS, Rajiv Grover, has commented that: ‘It’s business as usual in the Wild West and the message from the government is clear: roll up and feel free to have a stab’ [18]. Some improvements, however, have been made. The Royal College of surgeons in October 2016 launched new guidance for patients on cosmetic surgery to protect them from ‘aggressive marketing’ and ‘ruthless’ sales tactics and they expected to create a register of certified surgeons who are appropriately qualified to provide particular procedures.^4^ The General Medical Council also issued new guidance which sets out the standards they expect from doctors who provide cosmetic interventions, including stipulations to market their services responsibly, seek a patient’s consent themselves rather than delegate this to somebody else and consider patients’ vulnerabilities and psychological needs when making decisions with them about treatment options.^5^ While this presumably will not do anything to prevent poorly qualified doctors from offering their services as a cosmetic surgeon, it will, at least, allow prospective consumers of cosmetic surgery services to ascertain whether the surgeon in question is appropriately qualified. Hopefully therefore, we may anticipate a marginally better regulated collection of cosmetic surgery providers in the UK. Yet in spite of the expected improvement, we question whether these small measures go far enough. Moreover, even if safety is eventually improved through the creation of the cosmetic surgeons register, this would do little to address the cultural harms that cosmetic surgery perpetuates, particularly for women.

## Reforming the Regulation of Cosmetic Surgery in the UK?

Currently cosmetic surgery in the UK is regulated in the exactly same way as medically necessary surgery. As we have mentioned above, cosmetic surgery is defined as acceptable and legitimate medical practice (‘proper medical treatment’), alongside medically necessary or beneficial surgery. As a matter of public policy, the criminal law prohibits consensual harmful activities unless they can be justified because they are medically necessary, or carried out in pursuit of legitimate sporting activity.^6^ The only time that a doctor might fall under the scrutiny of the criminal law for harming a patient will be if that patient dies. If death occurs as a result of a medical blunder—and assuming the doctor did not intentionally kill the patient, for that would be murder—a charge of gross negligence manslaughter could follow. Essentially therefore, the reckless cosmetic surgeon who harms (but does not kill) a patient through performing ill-considered, inadvisable and negligent surgery will not be troubled by the criminal law. Where surgery is concerned, provided it is in the best interests of the patient and is carried out by a qualified healthcare professional, there is no question that it falls within the medical exception to the criminal law and is thus lawful. But having considered the risks we might ask when, if ever, is non therapeutic and potentially harmful surgery in a person’s best interests?

We have argued elsewhere that when patients suffer at the hands of cosmetic surgeons, who, driven by commercial profit, recklessly undertake risky and non-therapeutic surgery, the usual public policy justification for the medical exception is absent [12]. For this reason we have suggested that there should be a more significant role for the criminal law, through the use of the Offences Against the Person Act 1961, when serious harm is inflicted. Detailed consideration of the criminal law is beyond the scope of this article, other than to say that the main obstacle to a greater role for the criminal law is the medical exception, which surgeons rely upon to legitimise what might otherwise be harmful criminal conduct. The medical exception rests on assumptions that surgery is performed in the best interests of the patient because it is therapeutic, or it is in the interests of another (for example, when donating a kidney).^7^ The difficulty here is that there is sometimes a fine line between plastic surgery for therapeutic reasons and non-therapeutic cosmetic surgery. For example, consider breast augmentation surgery for reconstruction after a mastectomy due to cancer, which is evidently therapeutic, compared with breast enhancement to treat psychological issues with self-image. The latter may also be therapeutic in a sense, but it does nothing to treat the possible psychological causes for the lack of self-worth and it harms physical health via the surgery. While sometimes no clear line can be drawn, in our previous paper we define all non-therapeutic cosmetic surgery along the same lines as the NHS [12].

We have also suggested that even when the surgery goes well, the professional ethics of the surgeon in normalising such invasive interventions for cosmetic purposes have a harmful societal effect and so, for this reason also, cosmetic surgery should not be included within the definition of ‘proper medical treatment’ as a means for justifying it. Instead, much tighter regulation and sufficiently informed consent for all non-therapeutic cosmetic surgery should be required in order to legitimise its performance rather than by recourse to the medical exception. This could look like the model in France where, following the enactment of the Kouchner law 2002, regulation is much stricter, consent procedures are far more detailed, and additional safeguards regulating advertising and requiring a ‘cooling-off’ period, to allow the consumer to reflect on the decision, have been brought in [9, 20]. Moreover, when things do go wrong in French cosmetic surgery, as generally in French medicine, there is a much more significant role for the criminal law [16]. Latham was hopeful that following the Keogh Review, the British government might look to the French approach to inform legal change in the UK but unfortunately this now seems unlikely [20]. While the GMC have recently (April 2016) issued ethical ‘Guidance for doctors who offer cosmetic interventions’, which is a welcome development, in the absence of substantive legal reform we might expect little if any improvement within commercial provision.

For others, even improved regulation might not go far enough. Dennis Baker has argued, like us, that cosmetic surgery is harmful in a direct sense because it causes physical harm and in an indirect sense because it reinforces artificial celebrity or racist appearances as the preferred social norm [1]. For Baker better regulation would not significantly reduce either of those harms, and so he has argued that all significantly invasive cosmetic surgery should be regarded as a criminal offence and thus prohibited because it is inherently harmful. Baker also argues that consent cannot be used to justify such harms, commenting that: ‘The medical profession has hidden the criminal harm in unnecessary cosmetic surgery by dressing it up as genuine medicine’ [1]. For Baker, it should be criminalised because it involves wrongful harm. While we agree that the current approach is highly problematic, we do not agree that competent adults should be prevented from seeking lawful cosmetic surgery within the UK. A ban would simply drive such surgery underground and overseas where the dangers may be greater. Furthermore, as we have discussed above, although we share concerns that some women seeking cosmetic surgery may be viewed as victims of patriarchy, an informed choice to surgically enhance oneself may be viewed as a rational and positive life choice within the context of that individual’s circumstances.

Like Baker, McHale has focused on the law as it applies to the choices that children can take in respect of their bodies and cosmetic surgery. McHale asks whether regulation and/or outright criminalisation of certain cosmetic procedures concerning children and adolescents should now be considered and she pinpoints Queensland in Australia as an interesting and instructive example where such sanctions apply. However, as McHale notes, a key weakness within arguments concerning tighter regulation and criminalisation is that domestic law is constrained by what is now a global market in healthcare, which has become particularly significant in relation to cosmetic surgery. McHale asks; ‘would the adolescent and parent denied surgery in the UK simply hop on Eurostar or EasyJet and receive treatment elsewhere?’ [21]. Clearly it is highly likely that this question would be answered in the affirmative. Thus, in a global cosmetic surgery market, domestic regulation may be irrelevant to the growing tide of tourists who elect to seek cosmetic surgery services abroad. Domestic regulation could also drive an increase in such tourism if surgery becomes too expensive and difficult to access in the UK. This would evidently be an unwelcome development if it brought with it additional risks and harms, especially harms that would also be potentially costly to the NHS.

## Cosmetic Surgery Tourism: Risks and Harms

In order to consider the regulatory challenges within a global cosmetic surgery market, it is important to explore the evidence and risks regarding cosmetic surgery tourism. Such tourism, which might be defined as the movement of patients from one location to another to undertake aesthetic procedures, is a significant and growing area of medical tourism [2]. The UK’s annual International Passenger survey shows that approximately 100,000 UK citizens go abroad each year for medical treatment, which is projected to rise about 20% per year. Evidence from other jurisdictions is also illuminating. For example, cosmetic surgery tourists make up about 85% of Australian medical tourists [2]. In the UK figures are harder to find but a survey conducted by Treatment Abroad found that, including dental treatment and obesity surgery (for have cosmetic purposes), cosmetic procedures account for 60–70% of all medical tourism (42% excluding dental and obesity).

With respect to the top destinations for UK tourists for cosmetic surgery, we see that Poland (40%), then Spain, India, Tunisia, Czech Republic are the most significant markets.^8^ Some early studies have presented the surgery tourist as highly mobile, wealthy elites [8]. Other recent empirical research has in fact found that these consumers are far from wealthy, most often being lower middle class and working class women [14]. Confirming this, as mentioned earlier, it has been found in many studies that cost is the motivating factor influencing decisions to travel abroad (with the exception of China where travel was to seek out better quality surgery).

When a person elects to travel abroad in order to undergo cosmetic surgery, there are a number of reasons why the usual risks of surgery may be magnified, yet there is little clear evidence to suggest that cosmetic surgery abroad is necessarily dangerous. In order to explore the risks, we have identified the main concerns as follows: a primary concern is that it will often be more difficult to check that the clinic/hospital in a foreign destination is safe and reputable. In the UK the Care Quality Commission (CQC) regulates all such surgical providers and while there may be similar regulatory organisations and mechanisms in some countries this will vary significantly. Moreover, even where such regulatory agencies exist, the language barrier may make it difficult to access any relevant information.

Secondly and similarly, checking that a surgeon working outside the UK is appropriately qualified will often be more challenging. In the UK, although we await the register of certified cosmetic surgeons, it is at least possible to check a doctor’s registration with the General Medical Council (GMC). Whether any similar system operates abroad will depend upon the jurisdiction and, once again, the customer’s ability to access and understand any relevant information. Linked to these regulatory issues is the fact that UK providers and surgeons delivering private cosmetic surgery services must be appropriately insured in case of malpractice so that the patient may be compensated. Again, there may be a corresponding requirement in certain other countries but this is by no means universal and if something does go wrong, pursuing damages within a foreign legal system will invariably present greater challenges. While insurance may be available to safeguard such consumers and to enable them to pay legal costs should it be necessary, this will inflate the cost of seeking surgery abroad and thus some travellers will not obtain adequate insurance.

A further concern is that patients are usually required to pay for a package deal prior to travel. Informed consent, if it occurs at all, and the initial consultation may be superseded by agreeing to the treatment and, crucially, paying for both travel and treatment. Consequently people who have paid for all or part of the treatment (and travel) and then travelled abroad to the destination, will naturally feel reluctant to cancel the planned procedure in the event that the consultation and consent procedure cause them to reconsider the decision to have surgery. It also raises issues for after care in the case of complications. If the procedure has been paid for in advance the package may not include after care or if it does it such after care may not be accessible if the patient has flown back to their home country.

If the surgery goes well, the very concept of a ‘cosmetic surgery holiday’ carries dangers because traditional holiday activities, such as lying on a beach, swimming, sight-seeing and drinking alcohol, are potentially risky following surgery. Finally, and assuming one flies to the chosen destination, both surgery and air travel intensify the risk of deep vein thrombosis (DVT) and so flying home shortly after the surgery should be avoided. The British Association of Plastic, Reconstructive and Aesthetic Surgeons (BAPRAS) suggest that people should wait five to seven days after procedures such as breast augmentation or liposuction and seven to ten days after facial or abdominal surgery before flying home.^9^ Considering that most people elect to have cosmetic surgery abroad to minimise cost, the additional expense of an extended stay abroad will often compel people to fly home before they should, thus increasing their chances of suffering DVT. We can therefore see that the combination of all these additional risks, together with the possible language barrier and cultural differences within a health care context, make for a potentially dangerous experience.

But do these concerns translate into actual harm? And if so, is this costly to the NHS? Regarding cosmetic surgery, Jeevan and Armstrong conducted a survey for the British Association of Plastic, Reconstructive and Aesthetic Surgeons [15]. 203 out of 325 surgeons responded and of these, 76 (37%) had seen patients in the NHS with complications arising from overseas cosmetic surgery. In an audit of the pan-Thames region, 35 out of 65 consultants replied to requests about cosmetic surgery impacts [15]. Sixty per cent of those replying had seen complications and the majority of these cases (66%) were emergencies that required inpatient admission.

It is important to note that although the very real risks outlined above present compelling reasons to urge caution, other evidence of actual harm (especially beyond that of cosmetic surgery in the UK) paints a different picture. In a study by Hanefeld et al. [13] the costs and benefits of medical tourism to the NHS presented more positive figures, with few admissions. Holliday’s research found only 17% of their participants had complications and of those only 2% were serious [14]. The research showed that 97% of those participating in the study were happy with the outcome of the surgery and would recommend their surgeon to a friend [14]. The high levels of satisfaction are perhaps surprising considering that 17% of this group experienced complications following the surgery, with 9% requiring further treatment on their return home [14]. Clues to this response, however, can be elucidated from other information in the study, which suggests that those questioned did not undertake the surgery on a whim or with unrealistically optimistic expectations. Rather, they were ordinary people on modest incomes who took a long time to reach the decision to access cosmetic surgery abroad. This indicates that they were conscious of the inherent risks and even where the road to recovery included complications, provided the ultimate result was satisfactory, the risks were regarded as worth taking. Yet there are some problems with making too many positive conclusions from this data. There may also be cases where the NHS does not cover complications and thus patients do not present in the above studies. In addition, any levels of satisfaction clearly do not mitigate the costs to the NHS. Finally even 9% requiring NHS treatment is a significant financial burden to a stretched health service in terms of patient beds, delayed procedures and public health risks such as increased antimicrobial resistance stemming from the likely use of antibiotics with these patients and potential for introducing hospital infections from their stay in another hospital.

We might also consider how fears regarding services abroad may be inflamed by the rhetoric of national professional bodies naturally motivated to protect the national market. Note that the study by Jeevan and Armstrong was conducted for the British Association of Plastic, Reconstructive and Aesthetic Surgeons, who arguably have a vested interest in persuading prospective patients to seek treatment within the UK. Gimlin has argued that narrative strategies that discredit foreign providers are employed by organisations seeking to invoke fear in the consumer [11]. Gimlin provides an account of her own very positive experience of accessing health services abroad (in Costa Rica), before exploring the way in which cosmetic surgeons’ professional organisations [11] seek to influence perceptions about services abroad. Gimlin notes that while the warnings presented on the websites of these organisations are; ‘framed as ‘educational’ they also portray associations members’ services as better, safer and more public spirited than those of foreign practitioners.’ [11] In her study she found British organisations often drew on constructions of foreign providers as deceitful, unhygienic and primitive.

Notwithstanding the risks involved in seeking such surgery—even if these are exaggerated by domestic cosmetic surgeons—the other side of the argument is that the availability of affordable cosmetic surgery abroad enables more widespread access to services that have long been available to the wealthier few in society. Thus, the benefits of cosmetic surgery—improved appearance and self-image leading to the alleviation of psychological anxiety related to the physical body and greater happiness—are now available to more people. Accordingly, in a society where previously only the wealthy, or perhaps those sufficiently desperate and/or vulnerable and willing to endure severe financial hardship, were able to utilise the services of cosmetic surgeons, the phenomenon of affordable cosmetic surgery abroad might be seen to be egalitarian. If we recall arguments about cosmetic surgery ‘normalising perfection’, which may eventually result in an underclass of cosmetically unenhanced, then it might be argued that cosmetic surgery tourism provides new hope to those previously unable to afford such surgery.

Yet the counter argument is that by making cosmetic surgery even more readily available to wider groups of women, we are perpetuating and heightening the harmful cultural normalisation of enhanced beauty. So while such surgical tourism might democratise access to this form of enhancement, we would argue that this is not a positive development. Resisting the inequity of the normalisation of perfection by making it more accessible will only create more pressure to conform. The only way is to resist such normalisation is arguably to restrict such surgery, to deter surgeons from bad practice and to reduce the demand for it, but is this is possible?

## Regulating (Harmful) Cosmetic Surgery in a Global Context

How do we deal with the harms (both physical and cultural) that stem from domestic and foreign surgery? From the available evidence we might predict that tightening regulation, or even criminalising certain cosmetic surgery in the UK, would fuel demand for surgery abroad. The motivating factor for such travel is currently financial but it is probably accurate to forecast that if stricter regulation meant that accessing surgery in the UK became more difficult, ease of access to certain foreign services would become important. How much harm will stem from this depends upon the nature and scope of regulatory approaches in other jurisdictions. Many countries have a regulatory approach that is equal, or superior, to that of the UK. France, as we have mentioned, now takes a much more precautionary approach than the UK, with recent legislation which has tightened up practices in order to safeguard patients [20]. Yet the most popular venues seem to have a more relaxed approach to regulation. With respect to Europe wide regulation, the European Committee for Standardisation (CEN) has very recently produced a European Standard for Aesthetic surgery services within the 33 member countries.^10^ Speaking about the new standard, the chair of the group, Dr Johann Umschaden, an Austrian specialist surgeon stated:

Even if there are specific regulations in some EU Members States on aesthetic surgery, some of them are lacking in terms of hygienic, technical issues, or they don’t include a risk analysis. Recent reports on incidents in the context of aesthetic surgery emphasize the importance of this comprehensive European Standard which was developed through an open, inclusive, multi disciplinary and evidence based process [6].

The standard is, of course, voluntary and so does not compel providers to improve the quality and safety of their services. Prospective consumers can, however, select only those providers who sign up to the standard, thus improving their chances of having a safer experience. We may therefore view the CEN Standard as a step in the right direction towards a more uniform and better regulatory approach, at least in Europe, though only time will tell us whether the anticipated improvements occur.

However, CEN Standard does not cover all of the countries that are significant cosmetic surgery destinations for UK women seeking surgery and so perhaps a global regulatory response is necessary. Because the problems of normalisation and the unwelcome societal implications reach far beyond Europe, it seems that global regulation would serve an important function, however, there are obvious difficulties in constructing any global response. First, there are wide differences in attitudes towards acceptable levels of risk and gaps in commercial medical services. In addition global regulation will compete against drives to encourage and open new markets and the free flow of services. Moreover, in such a global market, cost will ultimately be the most significant driver and as we know, regulatory measures, however soft, are invariably expensive which would render them unattractive to many jurisdictions.

In view of the clear evidence of the risks in cosmetic surgery and regulatory inadequacy both domestically and globally, are we asking too much of the law in this context? We see merit in Sharon Cowan’s argument that we perhaps need to take a break from the legal in order to transform the social, or at least use the social and the legal together, to address the harms that cosmetic surgery arguably perpetuates [7]. Thus, rather than focusing solely on using a regulatory response to discourage harmful surgery, we should also be considering how we might challenge the culture that positions women in relation to the normalised representations of beauty. But the legal is not redundant. Tighter regulation in the UK, including using the criminal law against surgeons who cause harm when they proceed with risky surgeries, will not prevent people from seeking services abroad. However a domestic response would hopefully send out symbolic message that such surgery is potentially dangerous and should therefore be treated with great caution. Here the legal and social could work together in the way Cowan describes. While the impact of the law may not be direct or quick to change social perceptions of cosmetic surgery, in the long run, knowing that cosmetic surgery in the UK is restrictively regulated may alert people to the dangers of the practice they are about to undertake. In light of the constraints on applying effective legislation on a global level, this domestic response could be to the best hope foring the harms of the global cosmetic surgery industry. Our approach would prioritise changes in domestic regulation but accepts that, as Riles argues, we must resist relying solely on law to mould the social world around us and must use it as in combination with other strategies of transformation [23].

## Conclusion

We have considered the complex and sometimes conflicting evidence regarding domestic cosmetic surgery and the risks of seeking services abroad. Our research suggests that the very real physical and cultural risks of cosmetic surgery, wherever it is performed, coupled with the normalisation of the surgically enhanced female, means that stricter control via regulation is desirable. A global regulatory response is unlikely, especially for the UK during a time in which (post Brexit) we are retreating from cross border regulation. Thus we should pin our hopes on domestic regulation. Considering the precise forms of any such stricter regulation is beyond the scope of this paper, yet as a starting point we have suggested that the French approach, which regulates advertising, marketing, informed consent and which necessitates a cooling off period, would better safeguard consumers. Additionally, the assumption that all cosmetic surgery is medically justified—and so beyond the reach of the criminal law—simply because qualified doctors are performing it, should be reviewed in order to adopt a more nuanced approach. This, we have argued, should include recourse to the criminal law when patients are harmed in certain circumstances.

Tightening regulation may not prevent people seeking such treatment elsewhere but by changing the law and thus sending a clear message about the harms of such surgery, we suggest that it would alert at least some potential consumers to the dangers and make some people reconsider the wisdom of cosmetic surgery. Questioning the medical ethics of performing harmful, highly invasive surgery for purely aesthetic purposes and subsequently tightening the regulatory approach would deny cosmetic surgery the credibility and legitimation it currently receives. While such changes in domestic regulation may drive an increase in people seeking out foreign providers it may also have certain other indirect effects that would deter such surgery and thwart normalisation by delegitimising it. This would, we argue, make women think twice about seeking cosmetic surgery at home or abroad.

## Footnotes

1 For example, breast augmentation costs approximately £1972 in Poland compared to £3736 in the UK. See, ‘Cosmetic surgery abroad: Is it worth the risk?’ *The Guardian*, Matthew Jenkin, 4 August 2014.

2 See ‘PIP breast implant scandal: compensation ruling upheld’ The BBC, 21 January 2014, available at http://www.bbc.co.uk/news/world-europe-25831237 Accessed 10 August 2015.

3 According to the Medical Defence Union, growing numbers of patients are suing plastic surgeons over mistakes during operations designed to improve their appearance. See *MDU Journal*, Volume 27, Issue 2 November 2011. See also http://www.theguardian.com/society/2012/jan/08/patients-sue-plastic-surgeons-faulty. Accessed 22 October 2014.

4 See the Royal College of Surgeons webpages: http://www.rcseng.ac.uk/surgeons/surgical-standards/working-practices/csic/main-areas-of-work.

5 See General Medical Council Guidance on cosmetic interventions http://www.gmc-uk.org/guidance/ethical_guidance/28687.asp.

6 See for example *R v Brown* [1993] 2 All ER 75.

7 For a discussion see P Lewis,(2012) ‘The Medical Exception’, Current Legal Problems, 65: 355 Griffiths and Mullock, above n5.

8 See http://www.ssss.leeds.ac.uk/files/2012/11/Preliminary-Findings-Brochure.pdf.

9 See http://www.bapras.org.uk/public/patient-information/cosmetic-surgery/cosmetic-surgery-abroad.

10 See http://standards.cen.eu/dyn/www/f?p=204:110:0::::FSP_PROJECT:36242&cs=176A1D841118B7605821C83828D8A19A8. Accessed 10 August 2015.
